# Effect of *Sechium edule* var. *nigrum spinosum* (Chayote) on Telomerase Levels and Antioxidant Capacity in Older Adults with Metabolic Syndrome

**DOI:** 10.3390/antiox9070634

**Published:** 2020-07-18

**Authors:** Graciela Gavia-García, Juana Rosado-Pérez, Itzen Aguiñiga-Sánchez, Edelmiro Santiago-Osorio, Víctor Manuel Mendoza-Núñez

**Affiliations:** 1 Research Unit on Gerontology, FES Zaragoza, National Autonomous University of Mexico, Mexico City 09230, Mexico; ggg1501@hotmail.com (G.G.-G.); juanarosadoperez@comunidad.unam.mx (J.R.-P.); 2 Hematopoiesis and Leukemia Laboratory, Research Unit on Cell Differentiation and Cancer, FES Zaragoza, National Autonomous University of Mexico, Mexico City 09230, Mexico; itzen.aguiniga@zaragoza.unam.mx (I.A.-S.); edelmiro@unam.mx (E.S.-O.)

**Keywords:** metabolic syndrome, telomerase, oxidative stress, antioxidants, ageing, *S. edule*

## Abstract

Patients with metabolic syndrome (MetS) have a redox imbalance, due to a decay in antioxidant capacity. Oxidative stress (OxS) is considered an important modulator of telomere shortening and telomerase activity. One of the fruits that has been associated with an antioxidant effect is *Sechium edule* and although its properties are well established, there is only one exploratory study evaluating its effectiveness in patients with MetS. The present investigation is a much more robust and controlled study, including a placebo group. Hence, we determined the effect of consumption of the dried fruit powder (500 mg, three times per day) for three months. We measured effects on telomerase levels, antioxidant capacity, and markers for OxS. The study was performed in a sample of 75 older adults: placebo group (*n* = 30) and experimental group (*n* = 45) with the diagnosis of MetS according to the National Adult Treatment Panel of the National Cholesterol Program III (NCEP/ATP III) criteria. All markers were measured before and after three months of treatment. There was a statistically significant decrease in lipoperoxides and protein carbonylation with an increased superoxide dismutase (SOD), as well as sustained levels of telomerase in patients who consumed *Sechium edule*. Our findings suggest that consumption of this fruit has a hypoglycemic, hypotensive, and antioxidant effect, without altering telomerase levels, which could suggest better protection against telomere shortening.

## 1. Introduction

Metabolic syndrome (MetS) is considered one of the main public health challenges worldwide since it affects almost a quarter of the adult population [1]. It consists of a set of metabolic alterations, such as hypertension, hyperglycemia, dyslipidemia, and visceral obesity that predispose to the development of multiple chronic noncommunicable diseases, including type 2 diabetes mellitus (T2DM), cardiovascular and renal disease, and even the development of disability; these are all common within the elderly population.

MetS is also considered a model of accelerated aging [2], characterized by an increase in oxidative stress (OxS) and low-grade systemic inflammation that contribute to the aging process [3,4]. However, to counteract OxS, some molecules prevent a sequential and univalent reduction of molecular oxygen through enzymatic systems. In such a way, enzymatic systems prevent the formation of reactive oxygen species (ROS), an effect that is achieved by the conversion of these species into molecules that are less harmful to the cell, before they can react with other molecules [5].

The nature of aging has been associated with several theories, among which stands out is the finite replicative capacity of cells, where the telomere is shortened in every round of DNA replication [6]. Although, OxS is also considered an important modulator of telomere length [7]. However, to limit telomere attrition, cells express telomerase, a reverse transcriptase that maintains telomere length despite the synthesis of new DNA sequences. Therefore, telomeric length and the level and/or activity of telomerase enzyme can be considered as biological biomarkers involved in cell aging [8].

The presence of MetS warrants the use of several drugs such as hypoglycemics, statins, fibrates, etc., that are used for long periods, and with possible side effects; sometimes, the prescription of these medications ends up being inappropriate or potentially dangerous in elderly patients [9]. That is why it is still a necessity to find new treatments or interventions that decrease OxS and simultaneously regulate some of the metabolic abnormalities of MetS. In this regard, *Sechium edule* (chayote) is an edible plant of the family *Cucurbitaceae* with attributed nutritional, biofunctional, phytochemical, and pharmacological properties, which include hypotensive, anti-inflammatory, hypoglycemic, antioxidant, and lipogenesis inhibition properties. Even though those properties have been documented, there is only one exploratory study where the effectiveness of this plant has been evaluated in patients with MetS [10].

For this reason, the objective of this study was to determine the effect of consumption of *Sechium edule* (as dried fruit powder) on the levels of telomerase, antioxidant capacity, and OxS markers in older adults with MetS.

## 2. Materials and Methods

### 2.1. Design and Subjects

After obtaining prior authorization from the Bioethics and Biosafety Committee of the Faculty of Higher Studies of Zaragoza (FES-Z), National Autonomous University of Mexico (UNAM), with the agreement number 23/02-SO/2.4.2 (ISRCTN43215432), the study was carried out. The study population was recruited from an open convocation, where we specified the objectives of this study and the criteria for admission; it was intended for adults older than 60 years old, with overweight or obesity, and from Mexico City. The announcement was published and distributed via social networks. It should be noted that all procedures were executed following the Helsinki Declaration of the World Medical Association and with the informed consent of all participants.

The capsules of *Sechium edule* and placebo were formulated using good practices for manufacturing and made in the FES-Z. For the capsules with nutraceutical substance, we used biological material (*Sechium edule* var. *nigrum spinosum*) provided by the Interdisciplinary Group for the study of *Sechium edule* of México S.A. (GISeM). The fruits of *Sechium edule* were collected within horticultural maturity. Selected fruits were chopped in small pieces, set to dry at 40 °C and then pulverized (epidermis, thorns, and seeds). Particle size was standardized to obtain a fine powder. Rheological studies were performed on the powder to guarantee filling, content homogeneity, and long-term stability of the capsules for both active and placebo.

The subjects who were assigned to the placebo group (PG) were provided with capsules with identical characteristics to those that contained the nutraceutical substance, but with lactose monohydrate and pharmaceutical talc, both United States Pharmacopeia (USP) grade (Sigma, St. Louis, MO, USA). The treatment consisted of consuming three 500 mg capsules/day of *Sechium edule* or placebo (one before each meal) for three months. We carried out a double-blind quasi-experimental study performed in a population of 100 older adults, with an average age of 67 ± 6 years; all of them were diagnosed with MetS according to the criteria of the National Adult Treatment Panel of the National Cholesterol Program III (NCEP/ATP III): (i) waist circumference ≥102 for men or ≥88 cm for women; (ii) triglycerides ≥150 mg/dL; (iii) high-density lipoprotein cholesterol (HDL-C) <40 mg/dL in men or <50 mg/dL in women; (iv) blood pressure ≥130/85 mmHg; and (v) glucose >110 mg/dL. For the clinical diagnosis of MetS, at least three of the above five criteria must be present [11]. The population was randomly assigned to the PG (*n* = 50) or experimental group (EG; *n* = 50). However, during the first month, 10 people from the PG group dropped out the study. Later during the second month, another 15 subjects (10 from PG and 5 from EG) left as well because they did not experience an effect with the consumption of the treatment.

The following parameters: anthropometric measurements, blood pressure, biochemical analyses, thiobarbituric acid reactive substances (TBARS) assay, carbonylated proteins, superoxide dismutase (SOD), glutathione peroxidase (GPx) and catalase (CAT) activity, total antioxidant status (TAS), and telomerase concentration were measured at baseline and after three months of intervention.

### 2.2. Anthropometric Measurements

To appreciate the corporal dimensions, height and weight were determined. Subjects were weighed using a medical scale (Torino), wearing a clinical gown. For height determination, a wall stadiometer was used (SECA, Hamburg, Germany). To obtain a proper determination, subjects were required to step with heels together and head in contact with the stadiometer. Subjects kept their head upright on Frankfort’s plane, meaning that a horizontal imaginary line was drawn from the ear to the eye’s orbital area. Body mass index (BMI) was calculated using the weight ratio with the squared height (kg/m^2^). To determine the abdominal fat distribution of the subjects, the abdominal circumference was measured at the level of the navel without exerting pressure on the body using an asbestos measuring tape. The measurements were collected by trained personnel of the FES-Z [12].

### 2.3. Blood Pressure

The subjects’ blood pressure (BP) was measured on both arms, under fasting conditions using a mercury manometer. Osler’s technique was used to identify pseudo hypertension; trained personnel carried out the measurement [13].

### 2.4. Blood Sampling and Biochemical Analyses

Blood samples were obtained by venipuncture after a period of eight hours of fasting in vacuum tubes without anticoagulant to obtain serum for biochemical determinations (glucose, albumin, lipid profile, liver profile, and renal profile as well for telomerase concentration) with ethylenediaminetetraacetic acid (EDTA) as an anticoagulant for glycosylated hemoglobin (HbA1c) and with heparin for OxS tests. Heparinized samples were fractioned for the following determinations: activity of SOD and GPx from whole blood and plasma for TAS, CAT, lipid peroxidation (LPO), and carbonylated proteins. The techniques for SOD, GPx, CAT, TAS, protein carbonylation, and telomerase were performed microscale in multi-well plates, which were read in a Multiskan Go, version 1.00.40 (Thermo Scientific, Denver, CO, USA). Glucose, lipid profile, liver profile, renal profile, and albumin were determined by colorimetric techniques while HbA1c was determined by turbidimetry. All were performed with an automated Selectra Junior chemical-clinical analyzer (Vital Scientific, Dieren, Netherlands).

### 2.5. Plasma Thiobarbituric Acid Reactive Substances (TBARS)

This test is based on the reaction between malondialdehyde (MDA) and thiobarbituric acid (TBA) (0.11 mol/L) (Sigma, St. Louis, MO, USA) in phosphoric acid (H_3_PO_4_) (0.2 mol/L) (Sigma, St. Louis, MO, USA) which results in formation of the adduct TBA-MDA by oxygen removal and thereby produces a pink pigment with absorption at 535 nm. Possible amplification of the peroxidation during the assay is prevented by the addition of the antioxidant butylated hydroxytoluene (BHT) (Sigma, St. Louis, MO, USA) (12.6 mM) [14]. A mix of 200 µL of plasma, 25 µL of BHT, 200 µL of H_3_PO_4_, and 25 µL of TBA were incubated for 45 min at 90 °C. To stop the reaction, the mix was cooled in ice and later 500 µL of butanol (Sigma, St. Louis, MO, USA) were added alongside 50 µL of a saturated solution of sodium chloride (NaCl) (Sigma, St. Louis, MO, USA). The absorbance was read at 535 nm and 572 nm to correct for baseline absorption. The quantification was done using a calibration curve.

### 2.6. Carbonylated Proteins

Carbonylated proteins quantification was determined through the 2,4-dinitrophenylhydrazine (DNPH) test (Sigma, St. Louis, MO, USA). A mix of 20 µL of DNPH (10 mM in 0.5 M H_3_PO_4_) and 20 µL of plasma was incubated for 10 min at room temperature with continuous agitation; after that, 20 µL of 6 M sodium hydroxide (NaOH) were added. Finally, after 10 min of incubation at room temperature with continuous agitation, absorbance was measured at 450 nm against a blank sample. Due to the instability of dinitrophenylhydrazones in the alkaline medium, the period of incubation after the addition of NaOH was rigorously controlled [15]. Protein concentration was determined using a commercial Bradford reagent (Bio-Rad, Hercules, CA, USA) and a 1.41 mg/mL standard of bovine serum albumin (BSA). Samples were measured at 595 nm and the results expressed as protein mg/mL.

### 2.7. Whole Blood Superoxide Dismutase

This technique is based on the generation of superoxide radicals from xanthine and xanthine oxidase. The radicals formed react with 2-(4-iodophenyl)-3-(4-nitrophenol)-5-phenyl-tetrazolium chloride to form a red formazan dye, measured at 505 nm. The inhibition of this reaction allows to determine the activity of the enzyme. It was measured with a commercial kit (Randox Laboratories Ltd., Antrim, UK).

### 2.8. Whole Glutathione Peroxidase

The glutathione peroxidase catalyzes the oxidation of glutathione by cumene hydroperoxide in the presence of glutathione reductase and reduced nicotinamide adenine dinucleotide phosphate (NADPH). Oxidized glutathione is converted into the reduced form with the concomitant oxidation of NADPH to nicotinamide adenine dinucleotide phosphate (NADP^+^). This decrease in absorbance is measured at 340 nm (Randox Laboratories Ltd., Antrim, UK).

The SOD/GPx ratio and the antioxidant gap (AOGAP) were calculated. The AOGAP was calculated using the equation: AOGAP = (TAS − [(albumin (mmol) × 0.69) + uric acid (mmol)] [16].

### 2.9. CAT Activity

The activity of the CAT enzyme was determined by spectrophotometry, using hydrogen peroxide (H_2_O_2_) (Sigma, St. Louis, MO, USA) as a substrate. Plasma (10 µL) was incorporated into 190 µL of working solution (0.1 M phosphate buffer and 20 mM H_2_O_2_). Absorbance detection was performed at 240 nm, watching the decay of the H_2_O_2_ concentration every 15 s for 2 min [17]. Protein concentration was determined as mentioned earlier. The catalase activity was calculated using the equation: UCAT/g of protein = [(Abs 240 nm/0.0394) × (1000)]/mg/mL of protein].

### 2.10. Plasma Total Antioxidant Status (TAS)

This assay is based on the reaction of 2,2′-azino-bis (3-ethylbenzthiazoline-6-sulfonic acid) (ABTS) with metmyoglobin and H_2_O_2_ to generate the ABTS^+^ cation radical. This radical has a bluish-green coloration. The antioxidants present in the sample cause the suppression of this coloration and are proportional to their concentration (Randox Laboratories Ltd., Antrim, UK). The kinetics of the reaction were measured at 600 nm.

### 2.11. Serum Telomerase Levels

The concentration of telomerase enzyme (TE) was determined using a human TE ELISA kit (MyBioSource, San Diego, CA, USA) based on the principle of double-antibody sandwich technology, that is to say, TE antibody–TE antigen interactions (inmunosorbency) and purified horseradish peroxidase (HRP) colorimetric detection to detect TE antigen targets in serum samples at a wavelength of 450 nm. The optical density (O.D.) value was proportional to the concentration of the telomerase enzyme in the sample.

### 2.12. Statistical Analysis

Mean and standard deviation were calculated; the data were analyzed by ANOVA for repeated measures, using the statistical program IBM SPSS V 20 (Armonk, NY, USA). Chi-squared testing was used to compare proportions and percentage values were rounded. The statistical significance was *p* < 0.05. All the determinations were performed in duplicate.

## 3. Results

The percentages of the number of criteria met by the patients, in both PG and EG before and after treatment, are shown in Table 1. At the beginning of the treatment, both groups presented similar percentages in terms of the number of criteria. However, three months after treatment, there was a decrease in the presence of the number of criteria for both groups, although it was more evident in the EG. The treatment with *Sechium edule* improved in 53% of the subjects with MetS (<3 criteria) in contrast to 23% of the placebo. Based on the above, it is assumed that after treatment, 47% of EG participants continued with MetS for presenting at least three of its components compared to 77% of PG subjects. Thus, the consumption of *Sechium edule* reduces the presence of criteria for the clinical diagnosis of MetS.

Table 2 shows both clinical and anthropometric parameters of the experimental and placebo groups, before and after treatment. Regarding the anthropometric characteristics, the post-treatment EG showed a statistically significant decrease in body weight compared to the basal value as well as a BMI reduction. In addition, systolic blood pressure (SBP) decreased after treatment in both EG and PG. The same result was shown with diastolic blood pressure (DBP) for EG and PG. As for the results of waist circumference, no statistically significant post-treatment differences were observed in both EG and PG groups.

Regarding biochemical parameters, the EG showed a statistically significant decrease in glucose concentration after the treatment compared to the basal determinations as well as uric acid concentration, urea, creatinine and HbA1c determinations. On the other hand, the values of high-density lipoprotein cholesterol (HDL-C) increased after supplementation with *Sechium edule* in the EG (Table 3).

Table 4 shows the OxS determinations, where it is observed that LPO in the post-treatment EG showed a statistically significant decrease, as well as carbonylated proteins; while in the PG, protein carbonylation increased, together with telomerase levels. In addition, an increase in SOD enzymatic activity was observed in the EG group. On the contrary, CAT enzymatic activity and TAS levels showed a statistically significant decrease in the PG.

## 4. Discussion

It is estimated that 80% of elderly people suffer from a chronic non-infectious disease such as T2DM, hypertension, heart failure, cancer, and others. Some of these diseases are associated with MetS [18]. This syndrome has been in the spotlight in recent years for its elevated prevalence and for being considered a risk factor associated with diabetes and cardiovascular diseases [19].

One of the main components of the MetS is obesity, an entity whose treatment is complicated; however, there is evidence in obese animal models in which supplementation with *Sechium edule* for three months caused a reduction in body weight [20] that coincides with our findings, since we observed a weight reduction with a decrease in BMI after three months of supplementation. Nevertheless, regarding the effectivity of *Sechium edule* on patients with MetS, a six-week exploratory study did not show modifications of BMI [10]. This suggests that the supplementation period must be longer to significantly affect the weight and BMI. This effect probably is associated with the elevated insoluble fiber content of the chayote, such as hemicellulose (7.55 g/100 g dry weight) and cellulose (16.42 g/100 g dry weight), as well as lignin (0.23 g/100 g dry weight) [21]. In this sense, epidemiologic studies suggest that elevated fiber consumption reduces the risk of obesity, T2DM, and hypertension [22].

On the other hand, although a decrease in body weight and BMI was identified, this change was not observed in the waist circumference. This may be attributed to the fact that, despite the weight loss in the patients, the duration of the intervention was not enough to significantly modify the intra-abdominal fat, whose decrease correlated with the modification of the circulating lipids [23], an effect not observed in this study. Therefore, it is possible that a longer intervention time is required to achieve significant changes.

Another factor associated with MetS is hyperglycemia, which can lead to serious consequences at several levels, such as cardiovascular, neurological, renal, etc. [24]. In that sense, to reduce hyperglycemic development and its complications, *Sechium edule* extracts have been used in animal models, where it helped to normalize glucose tolerance capacity, lipid profile, and OxS [25,26]. Similarly, in the present study we found that blood glucose concentrations decreased significantly. The hypoglycemic effect of *Sechium edule* can be attributed to the presence of flavonoids like vicenin-2, vitexin, and apigenin 7-O-rutinoside that contribute to the previously reported properties [27].

Experimental evidence suggests that obesity and T2DM are associated with increased activity of tyrosine phosphatase 1β (PTP1β), a negative regulator of the insulin signaling pathway. In such a way, the juice of *Sechium edule* has shown an inhibitory activity over the expression of PTP1β, suggesting a signaling transduction system that can be attributed to the activity of anthocyanins and polyphenols that are present in fruit. Thus, *Sechium edule* consumption can be considered as a therapeutic agent with beneficial effects on insulin sensitivity, T2DM, and obesity [28,29].

On the other hand, it is estimated that 80% of people with MetS are hypertensive, which makes them susceptible to cardiac and cerebrovascular events [30]. *Sechium edule* is used as a therapeutic agent to control high arterial pressure by the antihypertensive and vasodilating effect [31,32]. In this regard, our results coincide since a diminution of BP in our experimental group was observed three months after treatment. Such an effect can be attributed to the presence of flavonoids with C-glycosidic, O-glycosidic or quercetin, with vasodilator and antidepressant effects [33]. In addition, it has been reported that Zucker rats with characteristics similar to MetS such as obesity, dyslipidemia, insulin resistance, and hypertension, improve with chronic high doses of quercetin [34]. It is notable that the positive effect of *Sechium edule* on the decrease in arterial pressure was like the post-treatment PG. This effect may suggest that placebos are not inactive, given the evidence that patients with hypertension often show lower arterial pressure values after placebo administration [35]. Moreover, the duration of the effect has been proved to be as prolonged as patients with an active treatment [36].

Likewise, we observed an increase in HDL-C levels in the post-treatment EG. This can be explained with the given pharmacological mechanisms of the extract of *Sechium edule*, which participates in the cholesterol homeostasis preservation. This effect can be associated with the antioxidant properties of this fruit, caused by its high levels of flavonoids, such as quercetin or naringenin [10], that also are suggested to improve HDL function and therefore cardiovascular health [37].

It is well documented that MetS has become a key factor in the risk of developing chronic kidney disease. These effects can be associated with insulin resistance, hypertension, dyslipidemia, inflammation, and OxS [38]. One study suggested a protective effect of *Sechium edule* leaves against nephrotoxicity in animal models, showing a reduction in serum markers of renal damage such as urea, creatinine, and uric acid [39]. These results are consistent with those obtained in the present study, where such renal parameters also showed a decrease after supplementation in patients with MetS. In consequence, results suggest that the active compounds in *Sechium edule*, like flavonoids, can improve kidney function [10].

HbA1c is an indicator of glycemic control, with the ability to reflect the cumulative glycemic history of the preceding two to three months [40], which is correlated to the risk of long-term complications of T2DM, and a risk factor for coronary disease and cerebrovascular accident. However, high-fiber fruit consumption is associated with a significant decrease in HbA1c and with a better function of pancreatic beta-cells [41,42,43,44]. We report a significant reduction of HbA1c in the EG post-treatment, which can be explained by the hypoglycemic effect of the fruit extract [44]. This is in contrast to what was reported in an exploratory study [10]; this suggests that the supplementation period needs to be longer to reduce HbA1c.

Regarding stress markers, our results demonstrate that the post-treatment PG showed a nearly nearly 50% increase in carbonylated proteins. Similar results have been obtained by other authors, independently of the presence or absence of T2DM in patients with MetS [45]. This type of damage can lead to protein structural modifications, dysfunctionality, and proteolysis susceptibility [46]. In the EG group, consumption of *S. edule* caused the diminution of carbonylated proteins levels after three months of treatment, a result attributed to the presence of phenolic compounds in this fruit.

Regarding lipoperoxides, our results show that the *Sechium edule* three-month treatment caused a 30% decrease, a result that coincided with a previous study in which there was a reduction of 17% after six weeks of treatment [10]. These findings suggest that the magnitude of the effect may be associated with consumption time; that is, intake of chayote gradually reduces the level of OxS in lipids. This reduction can be associated with the content of chlorogenic acid, caffeic acid, and quercetin [47], all of which have the capacity to interact with the antioxidant response element (ARE), favoring Nrf2 nuclear translocation, and inducing the transcriptional activity of genes that code for antioxidant enzymes like SOD, GPx, and CAT [48]. In this sense, we consistently observed an increase in SOD activity, a result not found in the exploratory study of six weeks of intervention, which again suggests that longer treatment time favors a better antioxidant response and is also consistent with the result observed in lipoperoxides [10]. This finding should be highlighted since it has been reported that there is a negative relationship between SOD activity and MetS components [49]. Likewise, regarding CAT, it has been reported that the red blood cells of patients with MetS do not fully process H_2_O_2_, leading to a higher risk of developing T2DM [50]. In this sense, in our results we found that there was a decrease of this enzyme in PG, while the levels were maintained in the group that consumed *Sechium edule*, which suggests that, although an increase such as that observed in SOD was not achieved, at least it was maintained, which can be considered as an improvement in the antioxidant system.

Regarding plasma antioxidants determined by TAS and the AOGAP [51], we observed that these markers presented a significant decrease in the PG while remaining in the EG. These results suggest that the consumption of *Sechium edule* improved the antioxidant response, both cellular and extracellular; we observed a decrease in antioxidant activity markers in the PG in contrast to the EG, where we observed the increase or maintenance of the antioxidant system and decrease in oxidation markers.

In this work, we observed that the PG showed a decrease in the levels at three months post-treatment. This indicates that antioxidant capacity is deficient, as well as the enzymatic antioxidant capacity mediated by CAT, which was identified by an increase in lipid and protein damage in this group.

It has been shown that telomere shortening during aging is accelerated by OxS [52]. Furthermore, it has been proved that an increase in the MetS components is associated with a shorter telomere length [53]. Nonetheless, telomerase helps to prevent telomere shortening. In this regard, individuals with chronic obstructive pulmonary disease (COPD) show higher levels of telomerase compared to smokers’ levels [54]. In addition, it is known that telomerase levels are higher in children that suffered burns and even higher in individuals with an amputated extremity [55]. In such a way, our results show an increase in telomerase levels in the post-treatment PG, as a possible mechanism that maintains the telomere, suggesting that the MetS per se leads to a gradual increase of the levels of this enzyme linked to greater DNA damage [56]. This suggests that, despite such increment, it is not enough to avoid telomere shortening, due to a possible deficiency in the repair mechanisms and/or by the inactivation of the transcription factors that sense the damage generated by the OxS in the DNA [57]; this leads to chromosomal instability, telomere dysfunction, cell death or premature cell senescence [55]. In this regard, it has been proposed that an increase of telomerase activity can lead to a senescent phenotype [58]. In other cases, telomerase overexpression leads to the development of malignant tumors. Thus, the link between MetS and cancer is becoming more evident, suggesting that the potential risk factors involved in the development of this process are obesity and T2DM, where the insulin-like growth factor-1 (IGF-1), alongside its intracellular signaling pathways, plays a key role in this kind of pathology [59]. Regarding the post-treatment EG, the levels of telomerase did not change, suggesting that the consumption of *Sechium edule* can counteract oxidative damage in patients with MetS, as shown with the lower levels of LPO and protein carbonylation. In addition, in terms of the number of criteria for MetS diagnosis, this treatment was shown to result in accumulation of fewer criteria such as body weight, BMI, and hypertension. In such a way, this study can help to understand the link between telomerase, OxS, and MetS.

Finally, it is important to point out limitations of this study, particularly the small sample size, and high dropout rate in the PG. However, our analysis of variance of repeated measures allows us to show the changes in the differences between the groups, even if the value of the quantified parameter is different in the baseline measurement. The groups were also not proportional by sex and age, so the influence of these variables could not be evaluated, and study follow-up was only performed once at three months. In this sense, it would be advisable to carry out a longitudinal study with more clinical evaluations and a crossover design.

## 5. Conclusions

Our findings show that the consumption of *Sechium edule* var. *nigrum spinosum* has a hypoglycemic, hypotensive, and antioxidant effect, without altering telomerase levels, which could suggest better protection for telomeric DNA avoiding telomere shortening. This result allows us to propose that the consumption of this fruit can be used to support the routine treatment of MetS, with the opportunity to improve the preventive strategies and/or slow progression of disease.

## Figures and Tables

**Table 1:** Percentage of patients with metabolic syndrome (MetS) who improved after treatment.

Placebo			Experimental	
Number of MetS Criteria	Baseline *n* (%)	Post-Treatment *n* (%)	Baseline *n* (%)	Post-Treatment *n* (%)
**2**	0 (0)	7 (23)	0 (0)	24 (53) *
**3**	19 (63)	18 (60)	28 (62)	16 (35) *
**4**	9 (30)	4 (13.3)	14 (31)	4 (9)
**5**	2 (7)	1 (3)	3 (7)	1 (1)
**Total**	30 (100)	30 (100)	45 (100)	45(100)

Data are expressed as percentages. ≥3 criteria = presence of MetS; <3 criteria = absence of MetS. Chi-squared test * *p* < 0.05.

**Table 2:** Anthropometric characteristics and blood pressure by group.

Parameter	Placebo *n* = 30	Experimental *n* = 45	*p*-Value
Body weight (kg)			
Baseline	78 ± 20	76 ± 15	0.04
Post-treatment	78 ± 20	75 ± 15 *	
BMI			
Baseline	31.8 ± 6.6	32.5 ± 5.5	0.03
Post-treatment	31.3 ± 6.6	32.0 ± 5.4 *	
Circumference of the waist (cm)			
Baseline	104.5 ± 15	103.0 ± 17	0.60
Post-treatment	102.4 ± 15	104.5 ± 11	
SBP (mmHg)			
Baseline	135 ± 12	131 ± 14	0.03
Post-treatment	127 ± 11 *	127 ± 12 *	
DBP (mmHg)			
Baseline	89 ± 8	85 ± 11	
Post-treatment	83 ± 9 *	80 ± 9 *	0.01

Data are expressed as means ± standard deviation. ANOVA of repeated measures test, significance level 95%, *p* < 0.05. BMI: body mass index; SBP: systolic blood pressure; DBP: diastolic blood pressure. * Statistical significance regarding baseline.

**Table 3:** Biochemical parameters pre- and post-treatment.

Parameter	Placebo *n* = 30	Experimental *n* = 45	*p*-Value
Glucose (mg/dL)			
Baseline	111 ± 57	137 ± 72	0.03
Post-treatment	121 ± 48	125 ± 64 *	
Cholesterol (mg/dL)			
Baseline	182 ± 40	205 ± 42	0.2
Post-treatment	191 ± 43	198 ± 43	
HDL-C (mg/dL)			
Baseline	33 ± 8	39 ± 11	0.001
Post-treatment	46 ± 9	48 ± 13 *	
Triglycerides (mg/dL)			
Baseline	178 ± 67	186 ± 80	0.1
Post-treatment	161 ± 58	171 ± 63	
Albumin (mg/dL)			
Baseline	4.4 ± 0.3	4.4 ± 0.3	0.1
Post-treatment	4.5 ± 0.3	4.3 ± 0.3	
Uric acid (mg/dL)			
Baseline	4.9 ± 1.3	4.8 ± 1.1	0.03
Post-treatment	5.1 ± 1.4	4.4 ± 1.3 *	
Urea (mg/dL)			
Baseline	36 ± 15	36 ± 11	0.01
Post-treatment	37 ± 14	32 ± 9 *	
Creatinine (mg/dL)			
Baseline	0.89 ± 0.27	0.86 ± 0.14	0.001
Post-treatment	0.91 ± 0.23	0.70 ± 0.25 *	
AST (U/L)			
Baseline	27.7 ± 9	26.3 ± 9	0.44
Post-treatment	26.4 ± 8	27.2 ± 12	
ALT (U/dL)			
Baseline	27 ± 11	31 ± 15	0.82
Post-treatment	27 ± 9	30 ± 18	
Total bilirubin (mg/dL)			
Baseline	0.56 ± 0.11	0.57 ± 0.23	0.21
Post-treatment	0.52 ± 0.15	0.60 ± 0.21	
Direct bilirubin (mg/dL)			
Baseline	0.19 ± 0.13	0.19 ± 0.09	0.6
Post-treatment	0.16 ± 0.06	0.18 ± 0.08	
HbA1c (%)			
Baseline	6.7 ± 1.9	7.1 ± 1.9	0.001
Post-treatment	6.3 ± 1.7	6.2 ± 1.8 *	

Data are expressed as means ± standard deviation. ANOVA of repeated measures test, significance level 95%, *p* < 0.05. HDL-C: high-density lipoprotein cholesterol; AST: aspartate aminotransferase; ALT: alanine aminotransferase; HbA1c: glycosylated hemoglobin. * Statistical significance regarding baseline.

**Table 4:** Markers of oxidative stress (OxS) and telomerase levels pre- and post-treatment.

Parameter	Placebo *n* = 30	Experimental *n* = 45	*p*-Value
Lipoperoxides (µmol/L)			
Baseline	0.24 ± 0.0	0.31 ± 0.1	0.001
Post-treatment	0.23 ± 0.1	0.22 ± 0.1 *	
Protein carbonylation (nmol/mg)			
Baseline	27 ± 5	31 ± 4.9	0.001
Post-treatment	41 ± 11	29 ± 4.1 *	
SOD (U/mL)			
Baseline	181 ± 13	182 ± 5	0.04
Post-treatment	187 ± 6	186 ± 6 *	
GPx (U/L)			
Baseline	6764 ± 2184	7289 ± 1887	0.83
Post-treatment	6444 ± 1651	7138 ± 2108	
SOD/GPx			
Baseline	0.28 ± 0.11	0.26 ± 0.59	0.4
Post-treatment	0.33 ± 0.12	0.29 ± 0.15	
CAT (U/mg)			
Baseline	3.43 ± 1.03	3.54 ± 1.82	0.02
Post-treatment	2.24 ± 0.66 *	3.05 ± 1.47	
AOGAP (µmol/L)			
Baseline	559 ± 182	361 ± 148	0.001
Post-treatment	312 ± 145 *	232 ± 174	
TAS (mmol/L)			
Baseline	1.23 ± 0.13	1.08 ± 0.19	0.01
Post-treatment	1.05 ± 0.21 *	0.99 ± 0.22	
Telomerase (ng/mL)			
Baseline	3.48 ± 1.01	4.33 ± 1.49	0.001
Post-treatment	4.20 ± 0.77 *	4.57 ± 1.51	

Data are expressed as means ± standard deviation. ANOVA of repeated measures test, significance level 95%, *p* < 0.05. SOD: superoxide dismutase; GPx: glutathione peroxidase; SOD/GPx: SOD/GPx ratio; CAT: catalase; AOGAP: antioxidant gap; TAS: total antioxidant status. * Statistical significance regarding baseline.

## Abbreviations

MetS	Metabolic syndrome
OxS	Oxidative stress
SOD	Superoxide dismutase
T2DM	Type 2 diabetes mellitus
ROS	Reactive oxygen species
FES-Z	Faculty of Higher Studies of Zaragoza
GISeM	Interdisciplinary Group for the study of *S. edule*
USP	United States Pharmacopeia
PG	Placebo group
EG	Experimental group
TBARS	Thiobarbituric acid reactive substances
GPx	Glutathione peroxidase
CAT	Catalase
TAS	Total antioxidant status
BMI	Body mass index
BP	Blood pressure
EDTA	Ethylenediaminetetraacetic acid
HbA1c	Glycosylated hemoglobin
LPO	Lipid peroxidation
MDA	Malondialdehyde
TBA	Thiobarbituric acid
H_3_PO_4_	Phosphoric acid
BHT	Butylated hydroxytoluene
NaCl	Sodium chloride
DNPH	2,4-dinitrophenylhydrazine
NaOH	Sodium hydroxide
AOGAP	Antioxidant gap
BSA	Bovine serum albumin
NADPH	Reduced nicotinamide adenine dinucleotide phosphate
NADP+	Nicotinamide adenine dinucleotide phosphate
H_2_O_2_	Hydrogen peroxide
ABTS	2,2’-azino-bis(3-ethylbenzothiazoline-6-sulfonic acid)
TE	Telomerase enzyme
HRP	Purified horseradish peroxidase
O.D.	Optical density
SBP	Systolic blood pressure
DBP	Diastolic blood pressure
HDL-C	High-density lipoprotein cholesterol
AST	Aspartate aminotransferase
ALT	Alanine aminotransferase
PTP1β	Protein tyrosine phosphatase 1β
ARE	Antioxidant response element
COPD	Chronic obstructive pulmonary disease
IGF-1	Insulin-like growth factor-1
